# Glycosaminoglycan-Mediated Downstream Signaling of CXCL8 Binding to Endothelial Cells

**DOI:** 10.3390/ijms18122605

**Published:** 2017-12-04

**Authors:** Rupert Derler, Bernd Gesslbauer, Corinna Weber, Elisabeth Strutzmann, Ingrid Miller, Andreas Kungl

**Affiliations:** 1 Antagonis Biotherapeutics GmbH, Strasserhofweg 77a, 8045 Graz, Austria; rupert.derler@edu.uni-graz.at; 2 Department of Pharmaceutical Chemistry, Institute of Pharmaceutical Sciences, University of Graz, Schubertstrasse 1, 8010 Graz, Austria; bernd.gesslbauer@uni-graz.at (B.G.); corinna.weber@krages.at (C.W.); elisabeth.strutzmann@gmail.com (E.S.); 3 Institute for Medical Biochemistry, University of Veterinary Medicine, Veterinärplatz 1, 1210 Vienna, Austria; ingrid.miller@vetmeduni.ac.at

**Keywords:** glycosaminoglycan, heparan sulphate, chondroitin sulphate, interleukin-8, downstream, signaling, proteomics, gene array

## Abstract

The recruitment of leukocytes, mediated by endothelium bound chemokine gradients, is a vital process in inflammation. The highly negatively charged, unbranched polysaccharide family of glycosaminoglycans (GAGs), such as heparan sulfate and chondroitin sulfate mediate chemokine immobilization. Specifically the binding of CXCL8 (interleukin 8) to GAGs on endothelial cell surfaces is known to regulate neutrophil recruitment. Currently, it is not clear if binding of CXCL8 to GAGs leads to endothelial downstream signaling in addition to the typical CXCR1/CXCR2 (C-X-C motif chemokine receptor 1 and 2)-mediated signaling which activates neutrophils. Here we have investigated the changes in protein expression of human microvascular endothelial cells induced by CXCL8. Tumor necrosis factor alpha (TNFα) stimulation was used to mimic an inflammatory state which allowed us to identify syndecan-4 (SDC4) as the potential proteoglycan co-receptor of CXCL8 by gene array, real-time PCR and flow cytometry experiments. Enzymatic GAG depolymerization via heparinase III and chondroitinase ABC was used to emulate the effect of glycocalyx remodeling on CXCL8-induced endothelial downstream signaling. Proteomic analyses showed changes in the expression pattern of a number of endothelial proteins such as Zyxin and Caldesmon involved in cytoskeletal organization, cell adhesion and cell mobility. These results demonstrate for the first time a potential role of GAG-mediated endothelial downstream signaling in addition to the well-known CXCL8-CXCR1/CXCR2 signaling pathways in neutrophils.

## 1. Introduction

The interaction between leukocytes and the endothelial cell surface is a key event in inflammatory processes. Glycosaminoglycans (GAG) at the endothelial cell surface are crucial mediators of this interaction [1]. This family of unbranched polysaccharides is found on all human cells as well as in the extracellular matrix and it consists of six different members, heparin (HP), heparan sulfate (HS), chondroitin sulfate (CS), dermatan sulfate (DS), keratan sulfate (KS) and hyaluronic acid (HA), which differ in their disaccharide building blocks. The most prevalent GAGs on the cellular surface are HS and CS. HS consists of repeating units of -d-GlcA-β-(1→4)-d-GlcNAc-α-(1→4)- with a variable degree of *N*-deacetylation/*N*-sulfation, *O*-sulfation and C5-epimerization; CS is made of is made of repeating -d-GlcA-β-(1→3)-d-GalNAc-β-(1→4)- units that can be modified by 2-*O*, 4-*O*, 6-*O*-sulfations and epimerization. The unique structural design, which in turn determines specific protein binding properties, is generated during biosynthesis by the concerted action of a complex set of enzymes [2,3]. During chain elongation, the nascent GAG chain is modified by an epimerase, converting GlcA into IdoA, and several sulfotransferases adding sulfate groups to distinct positions. Chain elongation and modification require an array of distinct enzymes for the HS and the CS pathway. The mature HS chain can also be edited by the action of endosulfatases and heparanase. Especially, the enzymes involved in the generation of the sulfation pattern exist in several isoforms with divergent activities, substrate specificities and tissue distribution. Modulation in GAG structure is therefore likely to be achieved, at least to some extent, by the differential regulation of expression of a certain repertoire of modifying enzymes.

Both GAG classes, HS and CS, are found covalently attached (*O*-linked) to core proteins, forming so called proteoglycans (PGs) of the syndecan (SDC) and glypican (GPC) family [4,5]. While the GPCs are linked to the membrane via C-terminal glycosylphosphatidylinositol anchors, the SDCs are the only transmembrane HS proteoglycans [6,7]. In mammals, four SDC isoforms are expressed (SDC 1 through 4) in a cell type, tissue and disease specific manner [8,9,10]. All SDC extracellular domains bear at least three HS chains close to their N-terminus, but to some extent also CS is attached at sites closer to the cell membrane [6,11,12]. The protein core components of PGs are synthesized in ribosomes to be then translocated to the rough ER where a xylosyltransferase initiates the synthesis of the linker tetrasacharide by adding a xylose to a serine residue of the protein core. Two galactose residues are subsequently added in the cis or medial Golgi to the Xyl by galactosyltransferase I and galactosyltransferase II. The fourth residue, completing the linker tetrassacharide, is a GlcA added by glucuronyltransferase I and occurs in the trans-Golgi, the final location for all subsequent reactions. The addition of the fifth saccharide determines whether the GAG chain becomes chondroitin sulfate (CS)/DS or HS/heparin. GAG type, length of the chain(s), conformational flexibility and particularly the specific GAG sequence/structure determine the biological function of the glycan part of the PG.

The structural features of these GAG chains enable SDCs to interact with a variety of soluble and insoluble molecules including growth factors [13,14], chemokines [15,16,17], extracellular matrix molecules [18,19], clotting factors [20,21] and proteins involved in lipid metabolism [22,23,24]. It is estimated that GAGs can bind to several hundred proteins [25,26,27]. GAG-protein interaction can lead to protection against proteolysis [28,29], mediation and changes in protein–protein interactions [30,31,32,33] and protein presentation on the endothelial cell surface [34,35]. Given their interaction with a vast number of proteins, as well as their multiple effects on these proteins, it comes as no surprise that GAGs are involved in a great number of physiologic events and malignancies.

CXCL8 is a member of the chemokine protein family, which encompasses small, generally basic chemotactic proteins. This chemokine is involved in numerous pathophysiological conditions including cancer [36], chronic obstructive pulmonary disease (COPD) [37] and rheumatoid diseases [38]. It is a well-known GAG-binding protein that is responsible for the recruitment of neutrophils to the site of inflammation by activating the chemokine receptors CXCR1 and CXCR2 [39]. Activation of these G protein coupled receptors leads to MAPK mediated cell activation mechanisms, such as cell migration, cell attachment and degranulation [40]. GAGs such as HS, which are integral part of cell surface proteoglycans (HSPGs), facilitate the formation of solid phase CXCL-8 gradients on endothelial surfaces, which is of central relevance in the multi-step process of leukocyte adhesion and endothelial transmigration [41,42,43]. In addition to CXCR1 and CXCR2, CXCL8 binds to DARC, a non-signaling chemokine receptor [44,45].

So far, it has not been investigated if CXCL8 binding to cell-surface HSPGs leads to intracellular signaling in endothelial cells of inflamed tissues. We have tested this hypothesis by investigating firstly the differential HSPG gene expression following TNFα stimulation, and secondly by proteomic analyses of protein expression following CXCL8 incubation of TNFα pre-stimulated human microvascular endothelial cells. Reshaping of the glycocalyx due to proteoglycan ectodomain shedding [46,47,48] and heparanase activity [49,50], which play an important role in vivo, were simulated by treatment with chondroitinase ABC and heparinase III. We found evidence that CXCL8-induced signaling via GAGs occurs in endothelial cells and that this influences the expression of proteins that are involved in cell adhesion and cell mobility.

## 2. Results and Discussion

### 2.1. Effect of TNFα on Proteoglycan Transcription and Expression in HMVECs

**Figure 1:**
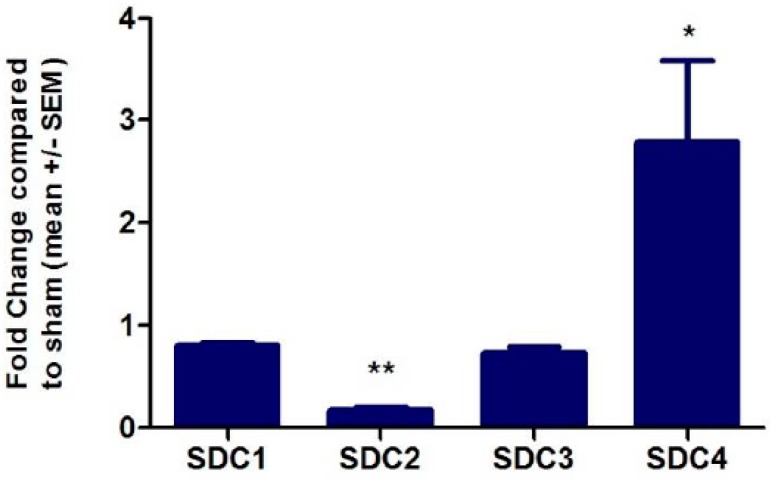
Fold-changes of SDC transcription in HMVECs (relative to glyceraldehyde 3-phosphate dehydrogenase (GAPDH) expression) following total RNA extraction after TNFα stimulation for 4 h, (*n* = 3; for further details see Materials and Methods). Statistical analysis by Student’s *t*-test: * *p*-value < 0.05; ** *p*-value < 0.005.

**Figure 2:**
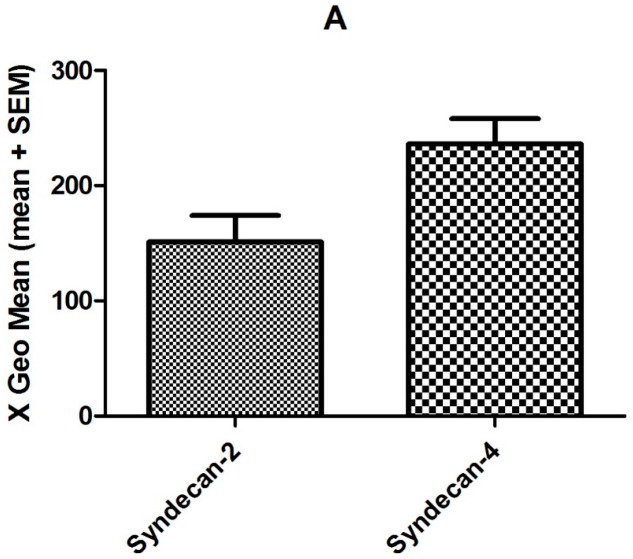

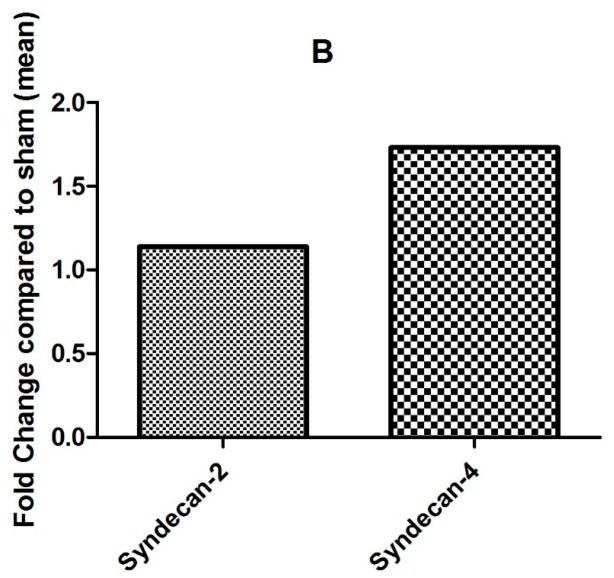
Flow cytometry analyses of endothelial SDC2 and SDC4 expression following TNFα stimulation. Shown are absolute expression values (**A**) and fold changes (**B**) compared to untreated cells for experimental details see Materials and Methods).

TNFα was added to the cell culture medium in order to screen for overall TNFα induced changes and changes in chemokine GAG co-receptor expression. RNA microarray screening revealed that changes in syndecan expression occurred, but that glypican expression remained unchanged (see Table S1 for a complete list of all changes). As an important internal control, the expression of CXCL8 was found to be 33-fold up-regulated following TNFα stimulation [51]. This corresponds to previous findings, see for example Reference [52]. RT-qPCR using SDC primers was applied to quantitate changes in SDC gene expression. For this means, human microvascular endothelial cells (HMVECs) were again stimulated with TNFα 50 ng/mL for four hours to induce an inflammatory response in vitro and to enable investigation of HS proteoglycan expression under inflammatory conditions. TNFα treatment resulted in a 2.7-fold increase in SDC4 transcription, while SDC2 expression was decreased 5.8-fold (see Figure 1). These findings were in accordance with the gene array measurements (see Table S1). On the protein level, SDC2 and SDC4 expression was examined by flow cytometry (see Figure 2). By this means, the expression level of SDC2 was found to be unchanged, whereas SDC4 expression was found to be 1.7-fold increased following TNFα stimulation. HS and/or CS chains of SDC4 are therefore highly likely to be the co-receptors of CXCL8 on endothelial cells in inflammatory conditions.

### 2.2. Effect of CXCL8 Treatment of Preinflamed HMVECs on Protein Expression

To investigate CXCL8 mediated downstream signaling, the chemokine was added to the cell culture medium (final conc. 50 nM) and changes in protein expression were detected. Exogeneous CXCL8 has been added to reach chemokine levels comparable to the in vivo situation. The amount of CXCL8 released by TNFα-stimulated endothelial cells alone is commonly much lower than the significantly higher concentrations of CXCL8 observed in inflammatory tissues, in which the chemokine is released also by other cells (mainly neutrophils, see Reference [53]). As internal controls, antibodies against CXCR1 and CXCR2 were added to rule out potential chemokine signaling via GPC receptors. On the protein expression level, differential expression via CXCR1- and CXCR2-independent signaling pathways included vimentin, a regulator of cell-adhesion and migration [54,55], and transgelin-2, both of which were significantly up-regulated (see Table 1). These changes in protein expression levels were the first hint towards specific signaling evoked by CXCL8 via GAGs in inflammatory settings (see Table S1 for a complete list of all expression level changes). Bioinformatic analyses showed four enriched protein clusters with rather unspecific annotations. For example annotation cluster 1 contained the GO terms poly(A)RNA binding with five proteins (cytoskeleton associated protein 4, heterogeneous nuclear ribonucleoprotein K, histone cluster 4 H4, ribosomal protein S18 and ribosomal protein S25) and RNA binding with three proteins (heterogeneous nuclear ribonucleoprotein K, ribosomal protein S18, ribosomal protein S25). Functional expression of adhesion molecules on TNFα-pre-inflamed endothelial cells was shown recently in flow chamber experiments using CXCL8 to recruit and adhere neutrophils [56].

### 2.3. Effect of CXCL8 Treatment on Protein Expression Levels of TNFα-Preinflamed, Chondroitinase- and Heparinase-Treated HMVECs

**Figure 3:**
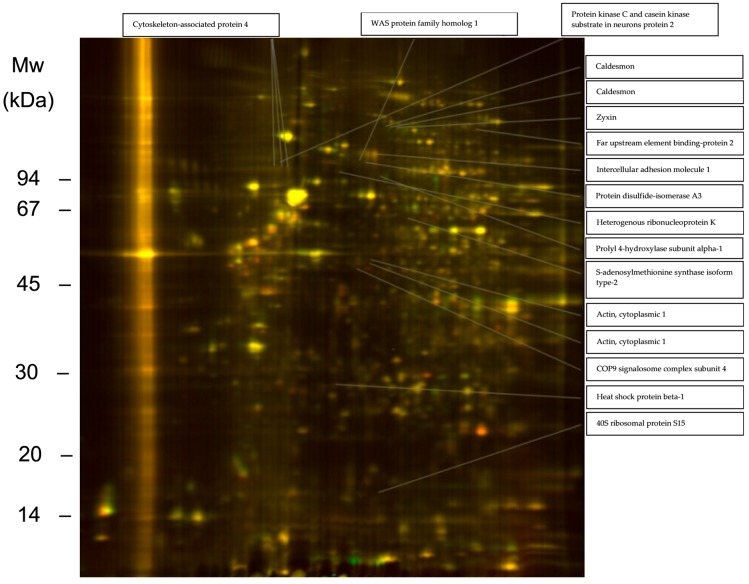
2D-DIGE gel of proteins from TNFα preinflamed, CXCL8 incubated cells treated with heparinase and chondroitinase (Cy3 stained, red) versus untreated controls (Cy5 stained, green). Proteins present in both samples appear in various shades of yellow depending on their relative quantity.

**Figure 4:**
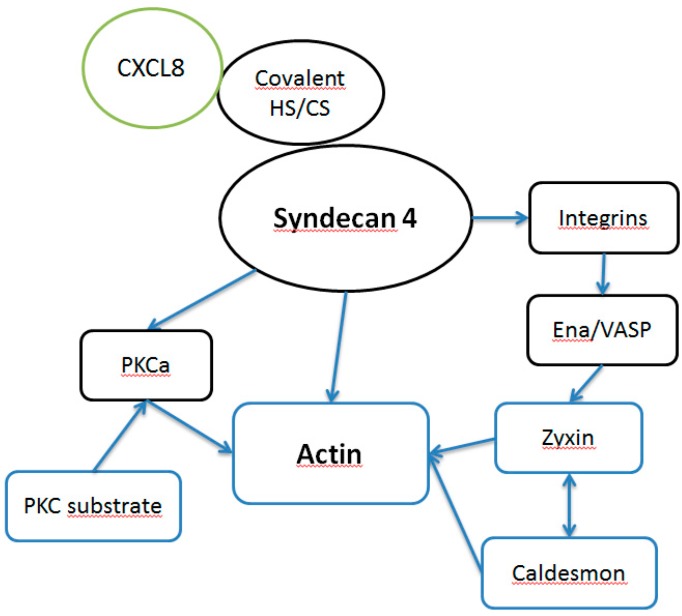
Scheme of potential GAG mediated CXCL-8 downstream signaling. Proteins in blue boxes were found to be differentially regulated upon CXCL-8 treatment.

Heparinase and chondroitinase were used to simulate glycocalyx reshaping of HS and CS and their respective proteoglycan core protein shedding that occur in vivo (for a list of endothelial proteins affected by chondroitinase and heparinase treatment alone see Table S1). CXCL8 stimulation of preinflamed, heparinase- and chondroitinase-treated HMVECs evoked a broader response in cellular proteome changes than in HMVECs without enzyme pre-treatment (see Table 2). Especially differential regulation of proteins directly involved in cell adhesion and/or cytoskeleton-associated proteins were observed under these conditions. Zyxin, caldesmon and cytoplasmic actin 1 expression were up-regulated, ICAM-1, cytoskeleton-associated protein 1 and prolyl 4-hydroxylase subunit alpha-1 expression were down-regulated (see Figure 3). Particularly proteins that are directly involved in actin assembly were found to be differently expressed. Zyxin, heat shock protein beta-1 (Hsp27), heterogenous ribonucleoprotein K (hnRNP K) mediate actin filament assembly [57,58,59]. Protein disulfide-isomerase A3 (PDIA3), Far upstream element binding-protein 2 (KSRP) and caldesmon are well known actin binding partners [60,61,62]. This suggests activation of a pathway influencing actin function. In Figure 4, potential GAG-mediated CXCL-8 downstream signaling pathways are presented. SDC4 that was found to be up-regulated after TNFα stimulus by qPCR is a proteoglycan known to affect the assembly of focal adhesions and actin stress fibers [63]. CXCL8 binding to SDC4 GAGs could influence this action, e.g., by mediating protein proteoglycan oligomerization. PKCa is a well-known regulator of actin function [64] that is functionally influenced by SDC4 [65]. SDC4 may affect PKCa action on actin in combination PKC substrate that was found to be up-regulated after CXCL8 binding to GAGs. Additionally, SDC4 acts on actin in a cooperative mode with integrins [66]. Zyxin contains an Ena/VASP binding domain that is crucial for actin dynamics [67]. Together with caldesmon this protein influences integrin mediated actrin stress fiber formation [68]. Taken together, GAG mediated downstream signaling could take effect directly via SDC4, an SDC4-integrin- or SDC4-PKCa-axis.

Functional annotation clustering of all differentially regulated proteins using DAVID Functional Annotation Tool (v8.6, see Section 3. Materials and Methods) underlines these findings. Six clusters were revealed of which five contained related GO terms, such as focal adhesion, cytoskeleton, cell–cell adhesion, cell junction and plasma membrane. For example annotation cluster 1 contained the eight proteins actin beta, heat shock protein beta-1, heterogenous nuclear ribonucleoprotein K, intercellular adhesion molecule 1, protein disulfide isomerase A3, protein kinase C and casein substrate in neurons, ribosomal protein S15 and zyxin. This cluster was annotated with GO terms focal adhesion, extracellular exosome and the sequence feature sequence variant. Together with abovementioned data from the literature, these findings show a clear involvement of the differentially expressed proteins in cellular structure and cell adhesion. Especially leukocyte migration requires dynamic cytoskeletal rearrangements at the endothelium. The observed proteomic changes imply a CXCL8 signaling that leads to reorganization of the cytoskeleton, a process crucially involved in the regulation of endothelial permeability in inflammation. Interestingly, expression of intracellular adhesion molecule 1 (ICAM-1), a major mediator of leukocyte adhesion that usually displays increased expression through inflammatory cytokines, was decreased, which adds further to the complexity of the GAG-chemokine interplay in inflammation. The fact that enzymatic reshaping of the glycocalyx led to an increased CXCL8 mediated signal underlines the mediatory function of GAGs at the cell surface. See Supplemental Material for a complete list of all changes.

## 3. Materials and Methods

### 3.1. Cell Culture

Human lung microvascular endothelial cells (HMVEC-l, Lonza, Basel, Switzerland) in the fourth passage were grown to 80% confluence in T75 flasks (Greiner Bio-One, Kremsmünster, Austria) containing 10 mL endothelial basal medium and growth supplements (Lonza). Where required, recombinant TNFα (Sigma-Aldrich, St. Louis, MO, USA) was added to a final concentration of 50 ng/mL and incubated for 10 h at 37 °C and 5% pCO_2_. TNFα incubation times and dosage have been optimized recently in our labs [69]. Where required, heparinase III (0.1 mU/mL, Iduron, Alderley, UK) and chondroitinase ABC (0.5 mU/mL, Sigma-Aldrich) were added to the culture medium after 30 min of incubation with TNFα. To rule out CXCL-8 signaling through CXCR1 and CXCR2 and binding to DARC/D6, 0.5 μg/mL of each anti-CXCR1, anti-CXCR2 and anti-DARC/D6 antibody (Santa Cruz, Dallas, TX, USA) were added to the medium. After incubation for 90 min, recombinant CXCL-8 (Antagonis Biotherapeutics GesmbH, Graz, Austria) was added to the medium at a final concentration of 50 nM. After incubation for 8 h, cells were washed with PBS twice, scraped into 2 mL PBS/EDTA and centrifuged in a 2 mL Eppendorf tube at 500× *g*. Residual cells in the plate were collected with 2 mL PBS/EDTA, added to the cell pellet and centrifuged again at 500× *g*. The supernatants were discarded and the cell pellets were stored at −80 °C until further use.

### 3.2. Whole Cell RNA Isolation

Total RNA was isolated from the cells using the total RNA isolation Kit (Sigma-Aldrich) according the manufacturer’s protocol. Quality and quantity of the isolated RNA was determined photometrically at 260 and 280 nm and by Bioanalyzer testing.

### 3.3. Gene Expression Analysis

Gene expression was investigated using the GeneChip^®^ Gene 1.0 ST Array System (Affymetrix, Santa Clara, CA, USA). cDNA synthesis from whole RNA, fragmentation and labelling was performed according to the Affymetrix^®^ GeneChip^®^ Whole Transcript (WT) Sense Target Labeling Assay Rev 5 protocol. For hybridization, the GeneChip^®^ Hybridization, Wash and Stain Kit was used according to the manufacturer’s protocol on a Fluidics Station 450. For scanning, the Affymetrix GCS3000 Scanner and the AGCC Command Console Software AGCC_3_1_1 was used. The Affymetrix Geneexpression Console v.1.1. was used for quality assessment. Data processing and filtering was done with the Partek Software v 6.4. For robust multi-chip analysis, background correction, quantile normalization across all chips in the experiment, log2 transformation and median polish summarization was done. Differentially expressed genes were identified by paired *t*-test using a *p*-value of 0.05 and a fold change threshold of 1.3.

### 3.4. Protein Isolation and Labeling

Cell pellets (approx. 6 × 10^6^ cells) were lysed in 75 to 100 µL of 30 mM Tris-HCl, 9 M urea, 4% CHAPS (*w*/*v*), pH 8.5. Solubilization was enhanced by two short incubations in a sonication bath for about 20 s each with intermittent cooling of the sample to 5 °C and one freeze-thaw cycle. Protein content was determined by a Coomassie G-250 protein-binding assay. 25 μg protein aliquots were labeled in triplicates with 200 pmol of CyDyes minimal dyes (GE Healthcare Life Sciences, Little Chalfont, UK) according to manufacturer’s protocol. Reverse labeling with Cy3 and Cy5 was performed for all samples in order to eliminate preferential labeling. Cy2 was used for the internal standard (a pool of all samples within one experiment), which was included on all gels. 

### 3.5. 2D Electrophoresis

Classical 2D electrophoresis was performed as previously published [70]. Samples were applied anodically to rehydrated laboratory made nonlinear IPGs pH 4 to 10 of 12 cm length and run on a Multiphor system (GE Healthcare, Little Chalfont, UK) for 20 kV/h. After 1D separation, strips were frozen until further use. For the second dimension, the strips were equilibrated and transferred to an SDS-PAGE gel (*T* = 10 to 15% linear gradient, *C* = 2.7%) according to Laemmli in a Hoefer SE 600 vertical electrophoresis chamber (Hoefer Scientific Instruments, Holliston, MA, USA). After 2DE, gels were scanned on a Typhoon 9400 imager and evaluated with DeCyder Software V5.02 (both GE Healthcare). The ratios between volumes of single spots in the samples and the corresponding spots in the internal standard were calculated. Statistic features in DeCyder were used for evaluation of 2-DE gels. Protein spots differentially expressed between samples were extracted from separate silver stained gels, using volume ratios of 1.5 as selection criteria. A modified silver staining protocol according to Heukeshoven [68] was used for detection. Gels were scanned with a Sharp JX-330 flatbed scanner. Differentially regulated spots were excised for mass spectrometry.

### 3.6. Mass Spectrometry

In-gel tryptic digestion, peptide extraction and nano-HPLC MS^2^ were performed as previously described [71]. Analysis of MS^2^ spectra with respect to peptide identity was routinely performed by applying both the GPM (Global Proteome Machine Organisation) and the SEQUEST (Thermo Finnigan, Waltham, MA, USA) search engines. In general a peptide was reliably identified only if the individual peptide scores XCorr were ≥2 for singly charged, ≥2.5 for doubly charged and ≥3.5 for triply charged peptides for SEQUEST, and if logE was ≤−2.5 for GPM. Peptides with logE scores between −1.5 and −2.5 were included only if the b and y ion series of the corresponding fragment showed at least 80% completeness. Only proteins identified with both search engines were considered. All peptides were blasted against the UniProt Knowledgebase.

### 3.7. RT-qPCR

2 µg of isolated whole cell RNA was reverse transcribed to cDNA using the High Capacity cDNA Reverse Transcription Kit (Applied Biosystems, Foster City, CA, USA) according to the manufacturer’s protocol. For the qPCR reaction, the Power SYBR Green PCR MasterMix was used according to the manufacturer’s protocol. Glycerinaldehyde-3-phosphate-dehydrogenase (GAPDH) was chosen as house-keeping reference gene. Following syndecan primers were used: Syndecan-1 (5′-AGGATGGAACTGCCAATCAG; 3′-ATCCGGTACAGCATGAAAGC), Syndecan-2 (5′-TCTGAGGCAGAAGAGAAGCTG; 3′-AGGATGAGGAAAATGGCAAA), Syndecan-3 (5′-ATACTGGAGCGGAAGGAGGT; 3′-TTTCTGGTACGTGACGCTTG), Syndecan-4 (5′-AACCACATCCCTGAGAATGC; 3′-AGGAAAACGGCAAAGAGGAT). The thermo-cycle and signal-detection was performed on an Applied Biosystems 7300 Real Time PCR System. The thermo-cycle consisted of an initial denaturation step (10 min at 95 °C), followed by 40 cycles of denaturation (15 s at 95 °C), primer annealing (30 s at 60 °C) and elongation (1 min at 72 °C). At the end, one final dissociation step (system default) was added. For data visualization and analysis, the 7000 system SDS software (Applied Biosystems, V 1.2.3) was used. Automatic Ct threshold and auto-baseline correction was chosen.

### 3.8. Flow Cytometry

Syndecan expression on the cell surface was determined using flow cytometry. HMVEC (Lonza). Cells were plated in 6-well plates (GBO, Kremsmünster, Austria), left untreated or stimulated with 50 ng/mL TNFα (PeproTech, Rocky Hill, NJ, USA) for 4 h at 37 °C/5% CO_2_, washed with PBS, detached using PBS/5mM EDTA for 20 min at 37 °C/5% CO_2_ and transferred to a FACS tube (Becton Dickinson, Franklin Lakes, NJ, USA).

Samples were then incubated with 0.25 μg/mL of the respective fluorophore-coupled antibody (monoclonal anti-human syndecan-4-allophycocyanin) from R&D Systems (Minneapolis, MN, USA) for 30 min at 4 °C. Isotype control was performed using 0.25 μg/mL APC Rat IgG2a IC (Becton Dickinson) antibody at the same labeling conditions. After incubation, samples were washed and fixed using BD Cellfix Buffer™ (Becton Dickinson). Data were acquired using a FACS Calibur (Becton Dickinson) with the software CellQuest™ (Becton Dickinson). Analysis was performed using FlowJo v7.6.5 (Treestar, Ashland, OR, USA).

### 3.9. Functional Annotation Clustering

DAVID Functional Annotation Tool v6.8 [1] was used to determine common protein characteristics. The tool carries out GO term annotation, protein functions, protein locations etc. calculates statistical significances by and clusters proteins in significantly groups. UniProt accessions of the differentially expressed proteins were entered and functional annotation clustering was performed against the Homo sapiens gene background using default parameters. The top five significant clusters were analyzed further. See Supplemental Material for the complete annotation and clustering data.

## 4. Conclusions

The obtained data revealed that the CXCL8-GAG-interactions are more complex than generally assumed. Transmembrane proteoglycans, which partly showed increased expression in inflammation, seem not only to facilitate a chemotactic gradient, but also to transduce CXCL8 mediated signals into the target cells. CXCL8 mediated reorganization of the actin cytoskeleton independent of conventional CXCR1 and CXCR2 pathways could be of relevance in the complex process of inflammation. We found that treatment of TNFα inflamed HMVEC with exogenous CXCL8 was associated with a rearrangement in the expression of cytoskeletal proteins, especially after treatment with heparinase and chondroitinase ABC. Reshaping of HS and CS with these enzymes in the glycocalyx lead to an amplification of changes in protein expression. This underlines findings that GAGs play a vital role in chemokine signaling, e.g., by binding proteins to the cellular surface and mediating chemokine interactions with other proteins. Modification of the GAG layer on the endothelial cell surface is thought to be an important mechanism in inflammatory processes in vivo. Usually GAG chains protrude further into the extracellular surroundings than common neutrophil adhesion receptors do. Common inflammation triggers like TNFα and IL-1 are known to regulate the expression of MMPs involved in glycocalyx reshaping and also in SDC ectodomain shedding. Additionally, heparanase is known for modifying the GAG composition on the cell surface and therefore their interaction with extracellular ligands. Thus, our results showed that remodeling of the GAG surface may lead to an enhanced direct chemokine exposure to receptors at the cell surface by decreasing the length of the GAG chains and capturing ligands more closely to different receptors. By ruling out conventional CXCR1 and CXCR2 signaling via antibody blockage, this leads to the conclusion that there might be a previously unknown GAG dependent CXCL8 signaling pathway that might control endothelial structure and permeability in inflammation via actin and actin binding proteins. We suggest that in inflammation an altered GAG profile determines the amount and type of chemokine interactions at the endothelial cell surface.

## Figures and Tables

**Table 1:** Differentially expressed proteins in CXCL-8 treated, pre-inflamed HMVECs.

Master No.	*t*-Test	Av. Ratio	Identified Protein	UniProtKB	MW (Da)
292	0.16	−1.59	Prelamin-A/C	P02545	74,380
373	0.086	−1.58	Alpha-2-HS-glycoprotein	P02765	40,098
389	0.25	−1.61	Cytoskeleton-associated protein 4 or	Q07065	66,097
			Heterogeneous nuclear ribonucleoprotein K	P61978	51,230
723	0.44	1.53	Vimentin	P08670	53,676
1020	0.003	1.59 *	Vimentin	P08670	53,676
1176	0.039	1.56 *	Transgelin-2	P37802	22,548
1194	0.063	1.73	40S ribosomal protein S18	P62269	17,708
1233	0.028	1.50 *	40S ribosomal protein S25/	P62851	13,791
			Prefoldin subunit 2	Q9UHV9	16,695
1294	-	1.76	Histone 4	P62805	11,360

* *p*-value < 0.05.

**Table 2:** Differentially expressed proteins in CXCL-8 treated, preinflamed, heparinase III and chondroitinase ABC treated HMVEC identified by proteomics.

Master No.	*t*-Test	Av. Ratio	Identified Protein	UniProtKB	MW (Da)
253	0.41	1.75	Zyxin	Q15942	62,463
254	0.43	1.61	Caldesmon	Q05682	93,251
257	-	1.81	Caldesmon	Q05682	93,251
259	0.040	1.64 *	Far upstream element binding-protein 2	Q92945	73,355
331	0.1	−2.46	Intercellular adhesion molecule 1	P05362	58,587
361	-	1.62	Protein kinase C and casein kinase substrate in neurons protein 2	Q9UNF0	56,046
374	0.15	−2.06	WAS protein family homolog 1	A8K0Z3	50,354
378	0.13	−1.88	Protein disulfide-isomerase A3	P30101	57,146
386	0.086	−1.56	Cytoskeleton-associated protein 4	Q07065	66,097
387	0.077	−1.62	Cytoskeleton-associated protein 4	Q07065	66,097
388	0.050	−1.77	Cytoskeleton-associated protein 4	Q07065	66,097
392	0.55	−1.87	Heterogenous ribonucleoprotein K	P61978	51,230
414	0.14	−2.09	Prolyl 4-hydroxylase subunit alpha-1	P13674	61,296
552	0.21	1.57	S-adenosylmethionine synthase isoform type-2	P31153	43,975
671	0.39	1.52	Actin, cytoplasmic 1/Nestin	P60709	42,052
				P48681	177,439
694	0.28	2.17	Actin, cytoplasmic 1	P60709	42,052
695	-	1.88	COP9 signalosome complex subunit 4	Q9BT78	46,525
1012	0.27	1.53	Heat shock protein beta-1	P04792	22,826
1208	-	−1.86	40S ribosomal protein S15	P62841	17,029

* *p*-value < 0.05.

## Supplementary Materials

Supplementary materials can be found at www.mdpi.com/1422-0067/18/12/2605/s1.

## Abbreviations

GAG	Glycosaminoglycan
HS	Heparan sulphate
CS	Chondroitin sulfate
PG	Proteoglycan

## References

[1] Götte M. (2003). Syndecans in inflammation. FASEB J..

[2] Esko J.D., Selleck S.B. (2002). Order out of chaos: Assembly of ligand binding sites in heparan sulfate. Annu. Rev. Biochem..

[3] Gesslbauer B., Rek A., Falsone F., Rajkovic E., Kungl A.J. (2007). Proteoglycanomics: Tools to unravel the biological function of glycosaminoglycans. Proteomics.

[4] Ihrcke N.S., Wrenshall L.E., Lindman B.J., Platt J.L. (1993). Role of heparan sulfate in immune system-blood vessel interactions. Immunol. Today.

[5] Rapraeger A., Jalkanen M., Endo E., Koda J., Bernfield M. (1985). The Cell Surface Proteoglycan from Mouse Mammary Epithelial Cells Bears Chondroitin Sulfate and Heparan Sulfate Glycosaminoglycans. J. Biol. Chem..

[6] Couchman J.R., Gopal S., Lim H.C., Norgaard S., Multhaupt H.A.B. (2015). Syndecans: From peripheral coreceptors to mainstream regulators of cell behaviour. Int. J. Exp. Pathol..

[7] Fico A., Maina F., Dono R. (2011). Fine-tuning of cell signaling by glypicans. Cell. Mol. Life Sci..

[8] Palaiologou M., Delladetsima I., Tiniakos D. (2014). CD138 (syndecan-1) expression in health and disease. Histol. Hostopathol..

[9] Theocharis A.D., Skandalis S.S., Tzanakakis G.N., Karamanos N.K. (2010). Proteoglycans in health and disease: Novel roles for proteoglycans in malignancy and their pharmacological targeting. FEBS J..

[10] Hacker U., Nybakken K., Perrimon N. (2005). Heparan sulphate proteoglycans: The sweet side of development. Nat. Rev. Mol. Cell Biol..

[11] Deepa S.S., Yamada S., Zako M., Goldberger O., Sugahara K. (2004). Chondroitin sulfate chains on syndecan-1 and syndecan-4 from normal murine mammary gland epithelial cells are structurally and functionally distinct and cooperate with heparan sulfate chains to bind growth factors. A novel function to control binding of midkine, pleiotrophin, and basic fibroblast growth factor. J. Biol. Chem..

[12] Hardingham T.E., Fosang A.J. (1992). Proteoglycans: Many forms and many functions. FASEB J..

[13] Zhang F., Zhang Z., Lin X., Beenken A., Eliseenkova A.V., Mohammadi M., Linhardt R.J. (2009). Compositional analysis of heparin/heparan sulfate interacting with fibroblast growth factor.fibroblast growth factor receptor complexes. Biochemistry.

[14] Asada M., Shinomiya M., Suzuki M., Honda E., Sugimoto R., Ikekita M., Imamura T. (2009). Glycosaminoglycan affinity of the complete fibroblast growth factor family. Biochim. Biophys. Acta.

[15] Pichert A., Samsonov S.A., Theisgen S., Thomas L., Baumann L., Schiller J., Beck-Sickinger A.G., Huster D., Pisabarro M.T. (2012). Characterization of the interaction of interleukin-8 with hyaluronan, chondroitin sulfate, dermatan sulfate and their sulfated derivatives by spectroscopy and molecular modeling. Glycobiology.

[16] Lau E.K., Paavola C.D., Johnson Z., Gaudry J.-P., Geretti E., Borlat F., Kungl A.J., Proudfoot A.E., Handel T.M. (2004). Identification of the glycosaminoglycan binding site of the CC chemokine, MCP-1: Implications for structure and function in vivo. J. Biol. Chem..

[17] Campanella G.S.V., Lee E.M.J., Sun J., Luster A.D. (2003). CXCR3 and heparin binding sites of the chemokine IP-10 (CXCL10). J. Biol. Chem..

[18] Battaglia C., Mayer U., Aumailley M., Timpl R. (1992). Basement-membrane heparan sulfate proteoglycan binds to laminin by its heparan sulfate chains and to nidogen by sites in the protein core. Eur. J. Biochem..

[19] Laterra J., Silbert J.E., Culp L.A. (1983). Cell surface heparan sulfate mediates some adhesive responses to glycosaminoglycan-binding matrices, including fibronectin. J. Cell Biol..

[20] Bisio A., Vecchietti D., Citterio L., Guerrini M., Raman R., Bertini S., Eisele G., Naggi A., Sasisekharan R., Torri G. (2009). Structural features of low-molecular-weight heparins affecting their affinity to antithrombin. Thromb. Haemost..

[21] Rosenberg R.D., Damus P.S. (1973). The purification and mechanism of action of human antithrombin-heparin cofactor. J. Biol. Chem..

[22] Ji Z.-S., Dichek H.L., Miranda R.D., Mahley R.W. (1997). Heparan Sulfate Proteoglycans Participate in Hepatic Lipaseand Apolipoprotein E-mediated Binding and Uptake of Plasma Lipoproteins, Including High Density Lipoproteins. J. Biol. Chem..

[23] Libeu C.P., Lund-Katz S., Phillips M.C., Wehrli S., Hernaiz M.J., Capila I., Linhardt R.J., Raffai R.L., Newhouse Y.M., Zhou F. (2001). New insights into the heparan sulfate proteoglycan-binding activity of apolipoprotein E. J. Biol. Chem..

[24] Ji Z.-S., Pitas R.E., Mahley R.W. (1998). Differential Cellular Accumulation/Retention of Apolipoprotein E Mediated by Cell Surface Heparan Sulfate Proteoglycans: Apolipoproteins E3 and E2 greater than E4. J. Biol. Chem..

[25] Kumar V., Hassan M.I., Tomar A.K., Kashav T., Nautiyal J., Singh S., Singh T.P., Yadav S. (2009). Proteomic analysis of heparin-binding proteins from human seminal plasma: A step towards identification of molecular markers of male fertility. J. Biosci..

[26] Ori A., Wilkinson M.C., Fernig D.G. (2011). A systems biology approach for the investigation of the heparin/heparan sulfate interactome. J. Biol. Chem..

[27] Gesslbauer B., Derler R., Handwerker C., Seles E., Kungl A.J. (2016). Exploring the glycosaminoglycan-protein interaction network by glycan-mediated pull-down proteomics. Electrophoresis.

[28] Ellyard J.I., Simson L., Bezos A., Johnston K., Freeman C., Parish C.R. (2007). Eotaxin selectively binds heparin. An interaction that protects eotaxin from proteolysis and potentiates chemotactic activity in vivo. J. Biol. Chem..

[29] Sadir R., Imberty A., Baleux F., Lortat-Jacob H. (2004). Heparan sulfate/heparin oligosaccharides protect stromal cell-derived factor-1 (SDF-1)/CXCL12 against proteolysis induced by CD26/dipeptidyl peptidase IV. J. Biol. Chem..

[30] Wu Z.L., Zhang L., Yabe T., Kuberan B., Beeler D.L., Love A., Rosenberg R.D. (2003). The involvement of heparan sulfate (HS) in FGF1/HS/FGFR1 signaling complex. J. Biol. Chem..

[31] Ecke S., Geiger M., Binder B.R. (1997). Heparin binding of protein-C inhibitor--analysis of the effect of heparin on the interaction of protein-C inhibitor with tissue kallikrein. Eur. J. Biochem..

[32] Xu D., Young J.H., Krahn J.M., Song D., Corbett K.D., Chazin W.J., Pedersen L.C., Esko J.D. (2013). Stable RAGE-heparan sulfate complexes are essential for signal transduction. ACS Chem. Biol..

[33] Nieto L., Canales A., Fernandez I.S., Santillana E., Gonzalez-Corrochano R., Redondo-Horcajo M., Canada F.J., Nieto P., Martin-Lomas M., Gimenez-Gallego G. (2013). Heparin modulates the mitogenic activity of fibroblast growth factor by inducing dimerization of its receptor. A 3D view by using NMR. Chembiochem Eur. J. Chem. Biol..

[34] Huber A.R., Kunkel S.L., Todd R.F., Weiss S.J. (1991). Regulation of transendothelial neutrophil migration by endogenous interleukin-8. Science.

[35] Netelenbos T., van den Born J., Kessler F.L., Zweegman S., Merle P.A., van Oostveen J.W., Zwaginga J.J., Huijgens P.C., Drager A.M. (2003). Proteoglycans on bone marrow endothelial cells bind and present SDF-1 towards hematopoietic progenitor cells. Leukemia.

[36] Zhu Y.M., Webster S.J., Flower D., Woll P.J. (2004). Interleukin-8/CXCL8 is a growth factor for human lung cancer cells. Br. J. Cancer.

[37] Kaur M., Singh D. (2013). Neutrophil chemotaxis caused by chronic obstructive pulmonary disease alveolar macrophages: The role of CXCL8 and the receptors CXCR1/CXCR2. J. Pharmacol. Exp. Ther..

[38] Szekanecz Z., Kim J., Koch A.E. (2003). Chemokines and chemokine receptors in rheumatoid arthritis. Semin. Immunol..

[39] Kuschert G.S.V., Coulin F., Power C.A., Proudfoot A.E.I., Hubbard R.E., Hoogewerf A.J., Wells T.N.C. (1999). Glycosaminoglycans Interact Selectively with Chemokines and Modulate Receptor Binding and Cellular Responses. Biochemistry.

[40] Murdoch C., Finn A. (2000). Chemokine receptors and their role in inflammation and infectious diseases. Blood.

[41] Proudfoot A.E.I. (2006). The biological relevance of chemokine-proteoglycan interactions. Biochem. Soc. Trans..

[42] Xu D., Esko J.D. (2014). Demystifying heparan sulfate-protein interactions. Annu. Rev. Biochem..

[43] Marshall L.J., Ramdin L.S.P., Brooks T., DPhil P.C., Shute J.K. (2003). Plasminogen Activator Inhibitor-1 Supports IL-8-Mediated Neutrophil Transendothelial Migration by Inhibition of the Constitutive Shedding of Endothelial IL-8/Heparan Sulfate/Syndecan-1 Complexes. J. Immunol..

[44] Horuk R., Chitnis C.E., Darbonne W.C., Colby T.J., Rybicki A., Hadley T.J., Miller L.H. (1993). A receptor for the malarial parasite Plasmodium vivax: The erythrocyte chemokine receptor. Science.

[45] Szabo M.C., Soo K.S., Zlotnik A., Schall T.J. (1995). Chemokine Class Differences in Binding to the Duffy Antigen-Erythrocyte Chemokine Receptor. J. Biol. Chem..

[46] Zhang Y., Wang Z., Liu J., Zhang Z., Chen Y. (2016). Suppressing Syndecan-1 Shedding Ameliorates Intestinal Epithelial Inflammation through Inhibiting NF-kappaB Pathway and TNF-alpha. Gastroenterol. Res. Pract..

[47] Nam E.J., Park P.W. (2012). Shedding of cell membrane-bound proteoglycans. Methods Mol. Biol..

[48] Manon-Jensen T., Itoh Y., Couchman J.R. (2010). Proteoglycans in health and disease: The multiple roles of syndecan shedding. FEBS J..

[49] Hammond E., Khurana A., Shridhar V., Dredge K. (2014). The Role of Heparanase and Sulfatases in the Modification of Heparan Sulfate Proteoglycans within the Tumor Microenvironment and Opportunities for Novel Cancer Therapeutics. Front. Oncol..

[50] Nadir Y., Brenner B. (2014). Heparanase multiple effects in cancer. Thromb. Res..

[51] Weber C. (2012). Investigating Chemokine Co-Receptor Interactions. Ph.D. Thesis.

[52] Cheng S.S., Lukacs N.W., Kunkel S.L. (2002). Eotaxin/CCL11 suppresses IL-8/CXCL8 secretion from human dermal microvascular endothelial cells. J. Immunol..

[53] Kobayashi Y. (2008). The role of chemokines in neutrophil biology. Front. Biosci..

[54] Dave J.M., Bayless K.J. (2014). Vimentin as an integral regulator of cell adhesion and endothelial sprouting. Microcirculation.

[55] Ivaska J., Pallari H.-M., Nevo J., Eriksson J.E. (2007). Novel functions of vimentin in cell adhesion, migration, and signaling. Exp. Cell Res..

[56] Adage T., Konya V., Weber C., Strutzmann E., Fuchs T., Zankl C., Gerlza T., Jeremic D., Heinemann A., Kungl A.J. (2015). Targeting glycosaminoglycans in the lung by an engineered CXCL8 as a novel therapeutic approach to lung inflammation. Eur. J. Pharmacol..

[57] Hirota T., Morisaki T., Nishiyama Y., Marumoto T., Tada K., Hara T., Masuko N., Inagaki M., Hatakeyama K., Saya H. (2000). Zyxin, a Regulator of Actin Filament Assembly, Targets the Mitotic Apparatus by Interacting with H-Warts/Lats1 Tumor Suppressor. J. Cell Biol..

[58] Pichon S., Bryckaert M., Berrou E. (2004). Control of actin dynamics by p38 MAP kinase—Hsp27 distribution in the lamellipodium of smooth muscle cells. J. Cell Sci..

[59] Nagano K., Bornhauser B.C., Warnasuriya G., Entwistle A., Cramer R., Lindholm D., Naaby-Hansen S. (2006). PDGF regulates the actin cytoskeleton through hnRNP-K-mediated activation of the ubiquitin E3-ligase MIR. EMBO J..

[60] LeBlanc T., Nemere L. (2014). Actin and Keratin are Binding Partners of the 1,25D3-MARRS Receptor/PDIA3/ERp57. Immunol. Endocr. Metab. Agents Med. Chem..

[61] Snee M. (2002). RNA trafficking and stabilization elements associate with multiple brain proteins. J. Cell Sci..

[62] Sobue K., Kanda K., Tanaka T., Ueki N. (1988). Caldesmon: A common actin-linked regulatory protein in the smooth muscle and nonmuscle contractile system. J. Cell. Biochem..

[63] Echtermeyer F., Baciu P.C., Saoncella S., Ge Y., Goetinck P.F. (1999). Syndecan-4 core protein is sufficient for the assembly of focal adhesions and actin stress fibers. J. Cell Sci..

[64] Larsson C. (2006). Protein kinase C and the regulation of the actin cytoskeleton. Cell. Signal..

[65] Keum E., Kim Y., Kim J., Kwon S., Lim Y., Han I., Oh E.-S. (2004). Syndecan-4 regulates localization, activity and stability of protein kinase C-alpha. Biochem. J..

[66] Saoncella S., Echtermeyer F., Denhez F., Nowlen J.K., Mosher D.F., Robinson S.D., Hynes R.O., Goetinck P.F. (1999). Syndecan-4 signals cooperatively with integrins in a Rhodependent manner in the assembly of focal adhesions and actin stress fibers. Proc. Natl. Acad. Sci. USA.

[67] Reinhard M., Jarchau T., Walter U. (2001). Actin-based motility: Stop and go with Ena/VASP proteins. Trends Biochem. Sci..

[68] Heukeshoven J., Dernick R. (1985). Simplified method for silver staining of proteins in polyacrylamide gels and the mechanism of silver staining. Electrophoresis.

[69] Strutzmann E. (2014). Investigating the Glycan-Mediated Chemokine Mode of Action. Ph.D. Thesis.

[70] Miller I. (2012). Application of 2D DIGE in animal proteomics. Methods Mol. Biol..

[71] Gesslbauer B., Poljak A., Handwerker C., Schuler W., Schwendenwein D., Weber C., Lundberg U., Meinke A., Kungl A.J. (2012). Comparative membrane proteome analysis of three Borrelia species. Proteomics.

