# Supplementary material for: Glycosaminoglycan-Mediated Downstream Signaling of CXCL8 Binding to Endothelial Cells
Source: Int J Mol Sci. 2017 Dec 4;18(12):2605. doi: 10.3390/ijms18122605 (PMC5751208; doi:10.3390/ijms18122605)
Supplement: Supplementary file 1 [file ijms-18-02605-s001.pdf]

## Supplemental Material

**Table S1.** Differentially expressed proteins in preinflamed, heparinase and chondroitinase treated HMVECs identified by proteomics.

| Master No. | *t*-test | Av. Ratio | Protein ID | UniProtKB | MW (Da) |
|---|---|---|---|---|---|
| **253** | 0.16 | 1.51 | Zyxin | Q15942 | 62463 |
| **374** | 0.42 | -1.53 | WAS protein family homolog 1 | A8K0Z3 | 50354 |
| **392** | 0.48 | -2.03 | Heterogeneous nuclear ribonucleoprotein K | P61978 | 51230 |
| **414** | 0.23 | -1.51 | Prolyl 4-hydroxylase subunit alpha-1 | P13674 | 61296 |
| **1176** | 0.071 | -1.68 | Transgelin-2 | P37802 | 22548 |

* $p$-value < 0.05.
